# Z-score of the log-transformed A Body Shape Index predicts low muscle mass in population with abdominal obesity: The U.S. and Korea National Health and Nutrition Examination Survey

**DOI:** 10.1371/journal.pone.0242557

**Published:** 2020-11-24

**Authors:** Shinje Moon, Yoon Jung Kim, Jae Myung Yu, Jun Goo Kang, Hye Soo Chung

**Affiliations:** Division of Endocrinology and Metabolism, Hallym University College of Medicine, Chuncheon, South Korea; Children’s Hospitals and Clinics of Minnesota, UNITED STATES

## Abstract

**Background and objective:**

Sarcopenic obesity is associated with a higher risk of cardiometabolic disease and mortality than either sarcopenia or obesity alone. However, no study has investigated body shape indices for the assessment of sarcopenia in obese populations. Thus, this study aimed to evaluate the accuracy of body shape indices to assess sarcopenia in nationally representative populations with abdominal obesity.

**Methods:**

Data from the U.S. National Health and Nutrition Examination Survey (U.S. NHANES) 1999–2006 and Korea NHANES (KNHANES) 2008–2011 were assessed. The association between Body Shape Index and sarcopenia was analyzed using a receiver operating characteristic curve. The Z-score of the log-transformed A Body Shape Index (LBSIZ) cut-off value was defined as that with the highest score of the Youden’s index. Changes in odds ratios (OR) for sarcopenia were investigated using restricted cubic spline (RCS) plots.

**Results:**

This study included 8,013 American and 4,859 Korean adults with abdominal obesity. The overall area under the curve (AUC) of LBSIZ for sarcopenia was 0.816 (95% CI: 0.794–0.838) in U.S. NHANES and 0.822 (95% CI: 0.799–0.844) in KNHANES, which was higher than that of the body roundness index, conicity index, and waist to height ratio (p with DeLong’s test <0.001). The cut-off values for the LBSIZ were 1.05 (sensitivity, 88.0%; specificity, 81.5%) for American men, 0.45 (sensitivity, 77.1%; specificity, 70.6%) for American women, 1.15 (sensitivity, 77.5%; specificity, 77.1%) for Korean men and 0.95 (sensitivity, 74.3%; specificity, 69.3%) for Korean women in the development groups. Comparable results were verified in validation groups. The RCS plot indicated that ORs for sarcopenia rapidly increased with an increase in the LBSIZ cut-off value.

**Conclusion:**

The increased LBSIZ could function as a reliable and cost-effective screening tool for assessing low muscle mass in populations with abdominal obesity.

## Introduction

The World Health Organization reported that 13% of the world’s population had obesity in 2016, and that the prevalence of obesity has dramatically increased over the last 30 years [1]. Obesity increases the risk of chronic diseases such as cardiovascular disease (CVD), diabetes, stroke, and cancer and is associated with approximately 4.8% of deaths worldwide [2]. Although obesity is defined as an excess of body fat, in clinical practice it is commonly assessed based on body mass index (BMI) [3]. However, BMI has a limitation for the estimation of the amount and distribution of body fat [4]. Considering that the abdominal deposition of adipose tissues is a major cause of CVD-related morbidity and mortality, waist circumference (WC) as an indicator of visceral adiposity has emerged as a complement to BMI [5–8]. Furthermore, WC was used to define metabolic syndrome instead of BMI in the National Cholesterol Education Program–Adult Treatment Panel III criteria (NCEP–ATP III criteria) [9]. However, BMI and WC both have limitations because they do not differentiate fat from lean mass.

Sarcopenia, which is the age-related loss of muscle strength and mass, is a well-known cause of disability, cardiometabolic disease, and mortality [10]. With aging, the body composition is modified based on an increase of visceral fat and a decline in muscle mass [11]. Visceral obesity and sarcopenia affect each other [10] and interact through adipokines and myokines [12]. Furthermore, they share common pathophysiological mechanisms, which include reduced physical activity, upregulated oxidative stress, inflammation, and insulin resistance [13, 14]. Sarcopenic obesity is a new category of obesity in the elderly population [13], which may be associated with a higher risk of metabolic disease, CVD, and mortality than sarcopenia or obesity alone [10, 15].

To simultaneously assess obesity and sarcopenia, body composition should be assessed because both BMI and WC are positively associated with fat mass and appendicular skeletal muscle [16]. Several imaging modalities are available to assess fat and muscle quantity or quality [17]. Computed tomography (CT) and magnetic resonance imaging (MRI) represent the gold standard for the diagnosis of sarcopenia based on the non-invasive measurement of muscle mass [18]. Furthermore, dual X-ray absorptiometry (DXA) is a more widely available modality and can reproducibly estimate fat and muscle mass in a few minutes [19]. However, highly trained experts are required to conduct these examinations, and these modalities are not commonly used because of the lack of portability and high equipment costs [18, 19]. Bioelectric impedance analysis (BIA), which is an indirect measurement of muscle mass based on whole-body electrical conductivity, is affected by hydration and recent physical activity; hence, more studies are needed to verify predictive equations for specific ethnic populations [19]. Furthermore, imaging tools are commonly unavailable in primary care environments, to which most senior adults with obesity or sarcopenia present [20]. Because of the increasing cost and complexity of medical care, simple and generally applicable anthropometry measurements of weight, height, and WC continue to provide prognostic information to medical practitioners and epidemiologists, and this information is comparable to that of more expensive and invasive tests [21]. Therefore, several anthropometric measurements to estimate body size and composition have been performed for the initial evaluation of sarcopenia [22, 23]. However, no study has investigated body shape indices with anthropometric data for the assessment of sarcopenia in obese populations.

Thus, this study aimed to evaluate the accuracy of the investigation of body shape indices for the assessment of sarcopenia in nationally representative populations with abdominal obesity from the U.S. National Health and Nutrition Examination Survey (U.S. NHANES) and Korea NHANES (KNHANES).

## Materials and methods

### Study population

**Fig 1:**
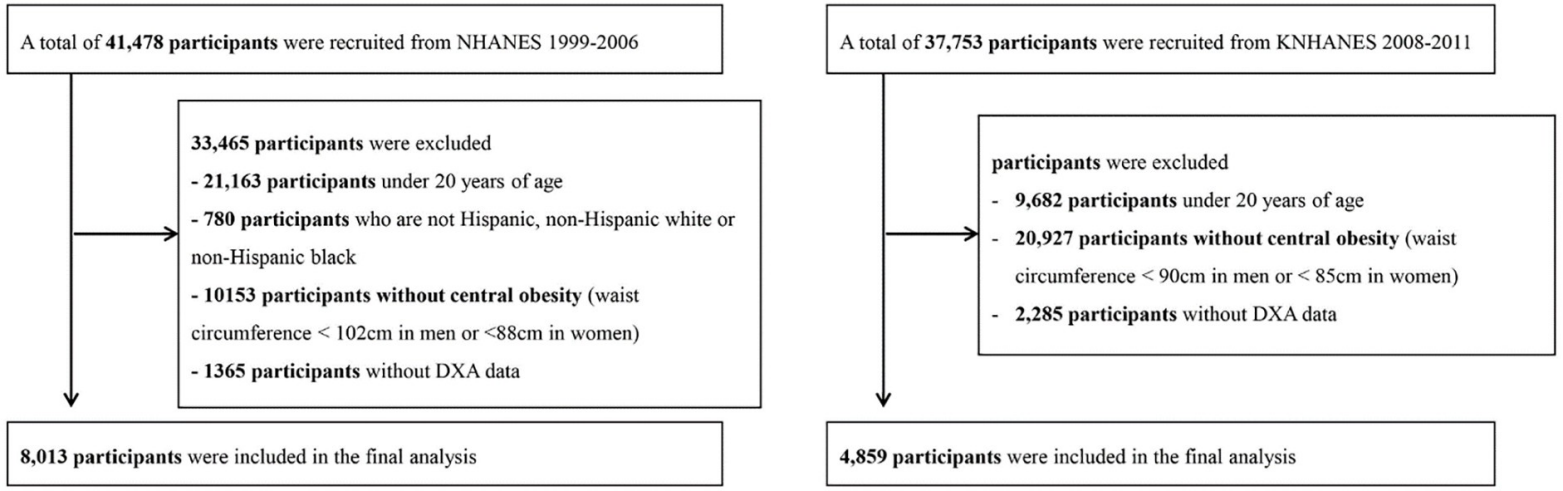
Flow chart of the final sample selection. NHANES, National Health and Nutrition Examination Survey.

Data from the U.S. NHANES from 1999 to 2006 and those from the KNHANES from 2008 to 2011 were assessed. The NHANES is a cross-sectional survey for samples that are representative of the United States population and is conducted biennially by the National Center for Health Statistics. The U.S. NHANES includes questionnaire-based personal interviews, physical examinations, and laboratory tests. KNHANES is an annual cross-sectional survey for samples that are representative of the Korean population and is conducted by the Korea Centers for Disease Control and Prevention (KCDC). The KNHANES comprises questionnaire-based personal interviews, physical examinations, and laboratory tests. In this study, among 41,478 participants of the U.S. NHANES, we included the data of 8,013 participants with abdominal obesity. We excluded those aged 20 years or younger, those without anthropometric data or DXA data, and those who were not Hispanic, non-Hispanic whites, or non-Hispanic blacks. After applying the exclusion criteria, except for race, for 37,753 KNHANES participants, finally, the data of 4,859 participants were included (Fig 1).

### Measurement of WC and body composition

WC in the U.S. NHANES was assessed by measuring the uppermost lateral border of the ilium using a flexible tape measure. Whole-body DXA examinations were conducted with a Hologic QDR 4500 A fan beam X-ray bone densitometer (Hologic Inc., Marlborough, MA, USA); furthermore, total and regional body compositions were analyzed with DXA scans. Additional details regarding the sample collection and examination method can be found in the NHANES Laboratory Procedures Manual [24].

WC in the KNHANES was assessed by measuring the midpoint of the lowest border of the rib cage and the upper lateral border of the iliac crest at the end of normal expiration. Whole-body DXA examinations were conducted using a QDR Discovery fan beam densitometer (Hologic, Inc., Bedford, MA, USA), and total and regional body compositions were analyzed with DXA scans. Additional details regarding the sample collection and examination method can be found in the guidelines of the KNHANES [25, 26].

### Definition of abdominal obesity and sarcopenia

For the American population, abdominal obesity was defined as a WC >102 cm in men and >88 cm in women based on the revised NCEP–ATP III criteria for metabolic syndrome [9]. For the Korean population, abdominal obesity was defined as a WC >90 cm in men and >85 cm in women according to the Korean Society for the Study of Obesity [27]. Appendicular skeletal mass was defined as the sum of the total lean mass, excluding the bone mineral content of both arms and legs, and the appendicular skeletal mass index (ASMI) was defined as the value obtained by dividing the appendicular skeletal mass by the square of the height (m). Sarcopenia for the American population was defined as an ASMI <7 kg/m^2^ in men or <5.5 kg/m^2^ in women according to the European Working Group on Sarcopenia in Older People 2 (EWGSOP2) [28], and sarcopenia for the Korean population was defined as an ASMI <7 kg/m^2^ in men or an ASMI <5.4 kg/m^2^ in women according to the Asian Working Group for Sarcopenia (AWGS) [29].

### Measurements of obesity parameters

Body roundness index (BRI), conicity index, and waist to height ratio (WHtR) were calculated based on the earlier suggested formulas, as shown below [30–32].

$$\text{BRI}:364.2-365.5\times \text{sqrt}[1-({\left(\text{WC}(\text{cm})/2\pi \right)}^{2}/{\left(0.5\times \text{Height}(\text{cm})\right)}^{2})]$$

ConicityIndex:WC(m)/[0.1093sqrt(Weight(kg)/Height(m))]

WHtR:WC(cm)/Height(cm)

The Z-score of the log-transformed A Body Shape Index (LBSIZ) was calculated based on the regression [ln(waist) = a0 + a1ln(weight) + a2ln(height) + δ]. The log-transformed Body Shape Index (LBSI) was calculated using log [waist/(exp(a0) × weight^a1^ × height^a2^], and LBSIZ was calculated using (LBSI-LBSI_mean_)/LBSI_standard deviation_. According to a previous study, a simple formula, derived using representative samples for each race, is provided in the supplementary Excel file format [16, 33], which is used in this study to calculate the LBSIZ values. Because LBISZ measures WC in relation to weight and height, higher values of LBSIZ reflect a greater abdominal obesity in those with the same value of height and weight, which implies a high level of visceral fat and a low level of muscle mass.

### Statistical analysis

Demographic characteristics, underlying diseases, anthropometric index, and blood test results were presented as means and standard deviations (SD) for continuous variables and prevalence (%) for categorical variables according to the presence of sarcopenia. The results were compared using an independent t-test and a Pearson’s chi-squared test. The relationship between the body shape indices and ASMI in each case was examined using a Pearson correlation coefficient. The area under the curve (AUC) for sarcopenia cases for each Body Shape Index was calculated using the ROC curve. DeLong’s test was used to statistically verify and ensure superiority of the AUC of the ROC curves when screening for sarcopenia [34]. We divided each dataset into subset for developing the LBSIZ cut-off value (NHANES 2003–2006 with 3,953 participant and KNHAENS 2010–2011 with 2,124 participants) and subset for validation (NHANES 1999–2002 with 4,060 participant and KNHANES 2008–2009 with 2,735 participants). The LBSIZ cut-off value was defined as the value observed when the Youden’s index value was the highest in the sensitivity-dominant area [35]. Using logistic regression analysis, the odds ratio (OR) for sarcopenia according to the LBSIZ cut-off value was derived with respect to sex. In addition, changes in ORs for sarcopenia with respect to sex were investigated using RCS plots with four knots. Statistical analysis was conducted using R ver. 3.1.0 (R Foundation for Statistical Computing, Vienna, Austria; www.r-project.org) and IBM SPSS Statistics ver. 24.0 (IBM Co., Armonk, NY, USA). P values <0.05 were considered statistically significant.

### Ethics statement

This study was approved by the Institutional Review Board of Kangnam Sacred Heart Hospital (IRB No. HKS 2017-07-007). All U.S. NHANES protocols were approved by the Research Ethics Review Board of the National Center for Health Statistics, U.S. Centers for Disease Control and Prevention (NCHS IRB/ERB Protocol Number: 1999–2004, Protocol #98–12; 2005–2010, Protocol #2005–06; 2011–2016, Protocol #2011–17), and all KNHANES protocols were approved by the Institutional Review Board of the KCDC (2007-02CON-04-P, 2008-04EXP-01-C, 2009-01CON-03-2C, 2010-02CON21-C, KCDC-2011-02CON-06-C). All participants volunteered and provided written informed consent before their enrolment. Their records were anonymized before being accessed by the authors. All analyses were performed according to approved guidelines and regulations.

## Results

This study was conducted with 8,013 American and 4,859 Korean adults with abdominal obesity. Among these, 375 Americans (4.7%) and 331 Koreans (6.8%) had sarcopenia. The baseline characteristics of the Americans and Koreans according to sex and the presence of sarcopenia are summarized in Tables 1 and 2, respectively. Common findings in both databases were that abdominally obese participants with sarcopenia were typically older and had a higher prevalence of hypertension and CVD despite having smaller WC and lower BMI. Among the obesity indices, LBSIZ and conicity index were significantly increased in the sarcopenia group. LBSIZ and conicity index were inversely correlated with ASMI and positively correlated with WC, whereas BRI and WHtR were both positively correlated with ASMI (Table 3).

**Table 1:** Characteristics of the participants in US NHANES according to sex and sarcopenia.

	Men			Women		
Variable	Without sarcopenia (N = 3024)	With sarcopenia (N = 101)	*p*	Without sarcopenia (N = 4614)	With sarcopenia (N = 274)	*p*
Age (years)	52.5 ± 16.4	72.3 ± 12.3	<0.001	51.2 ± 17.0	66.5 ± 14.7	<0.001
Ethnicity/race			<0.001			<0.001
Hispanic	731 (24.2%)	17 (16.8%)		1317 (28.5%)	88 (32.1%)	
Non-Hispanic White	1725 (57.0%)	82 (81.2%)		2092 (45.3%)	181 (66.1%)	
Non-Hispanic Black	568 (18.8%)	2 (2.0%)		1205 (26.1%)	5 (1.8%)	
Smoking (≥100 cigarettes in life)	1835 (60.7%)	76 (75.2%)	0.004	1823 (39.5%)	120 (44.0%)	0.166
Weight (kg)	101.8 ± 17.3	79.7 ± 7.6	<0.001	85.0 ± 17.8	63.7 ± 7.2	<0.001
Height (cm)	176.1 ± 7.6	172.7 ± 7.7	<0.001	161.0 ± 7.1	159.7 ± 7.3	0.005
Waist circumference (cm)	113.8 ± 10.6	107.8 ± 4.8	<0.001	104.3 ± 12.0	93.9 ± 4.9	<0.001
BMI (kg/m^2^)	32.8 ± 4.8	26.7 ± 2.1	<0.001	32.7 ± 6.2	25.0 ± 2.0	<0.001
LBSIZ	0.4 ± 0.7	1.8 ± 0.6	<0.001	-0.1 ± 1.0	1.1 ± 0.9	<0.001
BRI	6.61 ± 1.62	6.03 ± 0.89	<0.001	6.69 ± 1.98	5.21 ± 0.89	<0.001
Conicity index	1.38 ± 0.06	1.46 ± 0.05	<0.001	1.32 ± 0.08	1.37 ± 0.06	<0.001
WHtR	0.65 ± 0.06	0.62 ± 0.04	<0.001	0.65 ± 0.08	0.59 ± 0.04	<0.001
Systolic BP (mmHg)	129.1 ± 17.1	136.9 ± 21.8	0.003	128.1 ± 21.3	136.1 ± 25.4	<0.001
Diastolic BP (mmHg)	74.7 ± 13.4	68.8 ± 17.0	0.004	71.7 ± 12.1	67.6 ± 16.8	<0.001
Hypertension	1680 (59.9%)	79 (83.2%)	<0.001	2351 (55.6%)	182 (70.3%)	<0.001
FBG level (mg/dL)	114.6 ± 44.0	118.1 ± 40.1	0.583	109.5 ± 41.5	106.4 ± 32.5	<0.001
HbA_1C_ (%)	5.9 ± 1.2	5.9 ± 1.1	0.686	5.8 ± 1.2	5.6 ± 0.8	0.004
Diabetes Mellitus	648 (22.0%)	28 (28.3%)	0.178	836 (18.7%)	42 (15.8%)	0.276
Total cholesterol (mg/dL)	204.3 ± 43.4	200.8 ± 43.1	0.422	206.9 ± 42.1	218.2 ± 44.3	<0.001
Dyslipidemia	1444 (60.6%)	61 (66.3%)	0.326	2000 (58.0%)	161 (69.7%)	0.001
CVD*	356 (11.8%)	25 (24.8%)	<0.001	337 (7.3%)	39 (14.2%)	<0.001
ASMI	9.4 ± 1.4	6.6 ± 0.4	<0.001	7.5 ± 1.3	5.2 ± 0.3	<0.001

Data are presented as the means ± SD or number (%).

*Participants who had either angina pectoris, coronary heart disease, myocardial infarction, congestive heart failure, or cerebrovascular disease.

BMI, body mass index; LBSIZ, z-score of the log-transformed A Body Shape Index; BRI, Body Roundness Index; WHtR, waist to height ratio; BP, blood pressure; FBG, fasting blood glucose; HbA1c, hemoglobin A1c; CVD, cardiovascular disease; ASMI, Appendicular skeletal mass index.

**Table 2:** Characteristics of the participants in KNHANES according to sex and sarcopenia.

	Men			Women		
Variable	Without sarcopenia (N = 1935)	With sarcopenia (N = 158)	*p*	Without sarcopenia (N = 2593)	With sarcopenia (N = 173)	*p*
Age (years)	50.8 ± 14.7	66.3 ± 12.5	<0.001	56.9 ± 14.2	65.9 ± 12.6	<0.001
Smoking (≥100 cigarettes in life)	1548 (80.4%)	128 (82.1%)	0.696	234 (9.1%)	21 (12.7%)	0.163
Weight (Kg)	80.1 ± 9.4	68.4 ± 6.1	<0.001	66.5 ± 8.8	57.2 ± 5.9	<0.001
Height (cm)	170.7 ± 6.5	167.5 ± 6.0	<0.001	155.7 ± 6.3	152.4 ± 6.3	<0.001
Waist circumference (cm)	95.5 ± 5.2	93.5 ± 3.3	<0.001	91.8 ± 5.9	89.2 ± 3.8	<0.001
BMI (kg/m2)	27.4 ± 2.4	24.4 ± 1.6	<0.001	27.4 ± 2.9	24.6 ± 2.1	<0.001
LBSIZ	0.6 ± 0.8	1.8 ± 0.7	<0.001	0.6 ± 0.9	1.5 ± 0.9	<0.001
BRI	4.58 ± 0.79	4.55 ± 0.59	0.436	5.25 ± 1.02	5.15 ± 0.78	0.122
Conicity index	1.28 ± 0.05	1.34 ± 0.05	<0.001	1.29 ± 0.06	1.34 ± 0.06	<0.001
WHtR	0.56 ± 0.03	0.56 ± 0.03	0.544	0.59 ± 0.04	0.59 ± 0.03	0.182
Systolic BP (mmHg)	125.7 ± 15.5	129.6 ± 16.6	0.003	126.6 ± 18.1	129.5 ± 18.8	0.040
Diastolic BP (mmHg)	82.4 ± 10.6	78.4 ± 11.1	<0.001	78.3 ± 10.2	77.6 ± 9.9	0.373
Hypertension	969 (50.4%)	109 (69.4%)	<0.001	1308 (50.8%)	104 (61.9%)	0.007
FBG level (mg/dL)	105.8 ± 26.6	111.9 ± 34.1	0.038	104.6 ± 26.9	101.6 ± 24.9	0.186
HbA_1C_ (%)	6.7 ± 1.3	6.7 ± 1.2	0.791	6.8 ± 1.4	6.8 ± 1.4	0.872
Diabetes Mellitus	307 (16.9%)	38 (27.9%)	0.002	445 (18.7%)	29 (19.9%)	0.800
Total cholesterol (mg/dL)	194.4 ± 36.7	184.7 ± 35.8	0.002	199.1 ± 36.8	205.4 ± 38.8	0.042
Dyslipidemia	292 (16.1%)	19 (13.9%)	0.578	575 (24.0%)	43 (29.5%)	0.167
CVD*	101 (5.2%)	32 (20.4%)	<0.001	168 (6.5%)	17 (10.1%)	0.099
ASMI	8.4 ± 0.8	6.6 ± 0.3	<0.001	6.5 ± 0.7	5.1 ± 0.3	<0.001

Data are presented as the means ± SD or number (%).

*Participants who had either angina pectoris, coronary heart disease, myocardial infarction, congestive heart failure, or cerebrovascular disease.

BMI, body mass index; LBSIZ, z-score of the log-transformed A Body Shape Index; BRI, Body Roundness Index; WHtR, waist to height ratio; BP, blood pressure; FBG, fasting blood glucose; HbA1c, hemoglobin A1c; CVD, cardiovascular disease; ASMI, Appendicular skeletal mass index.

**Table 3:** Correlation of obesity indices with ASMI and waist circumference.

Group	KNHANES		NHANES	
	ASMI	WC	ASMI	WC
Men				
LBSIZ	-0.600**	0.168**	-0.626**	0.145**
BRI	0.021	0.496**	0.137**	0.802**
Conicity index	-0.386**	0.508**	-0.422**	0.486**
WHtR	0.160**	0.790**	0.135**	0.789**
Women				
LBSIZ	-0.498**	0.141**	-0.505**	0.135**
BRI	0.116**	0.589**	0.244**	0.836**
Conicity index	-0.272**	0.515**	-0.286**	0.504**
WHtR	0.254**	0.837**	0.239**	0.829**

* p value <0.05;

** p value <0.001.

ASMI, Appendicular skeletal mass index; LBSIZ, z-score of the log-transformed A Body Shape Index; BRI, Body Roundness Index; WHtR, waist to height ratio.

### Association between the obesity indices and sarcopenia in individuals with abdominal obesity

**Fig 2:**
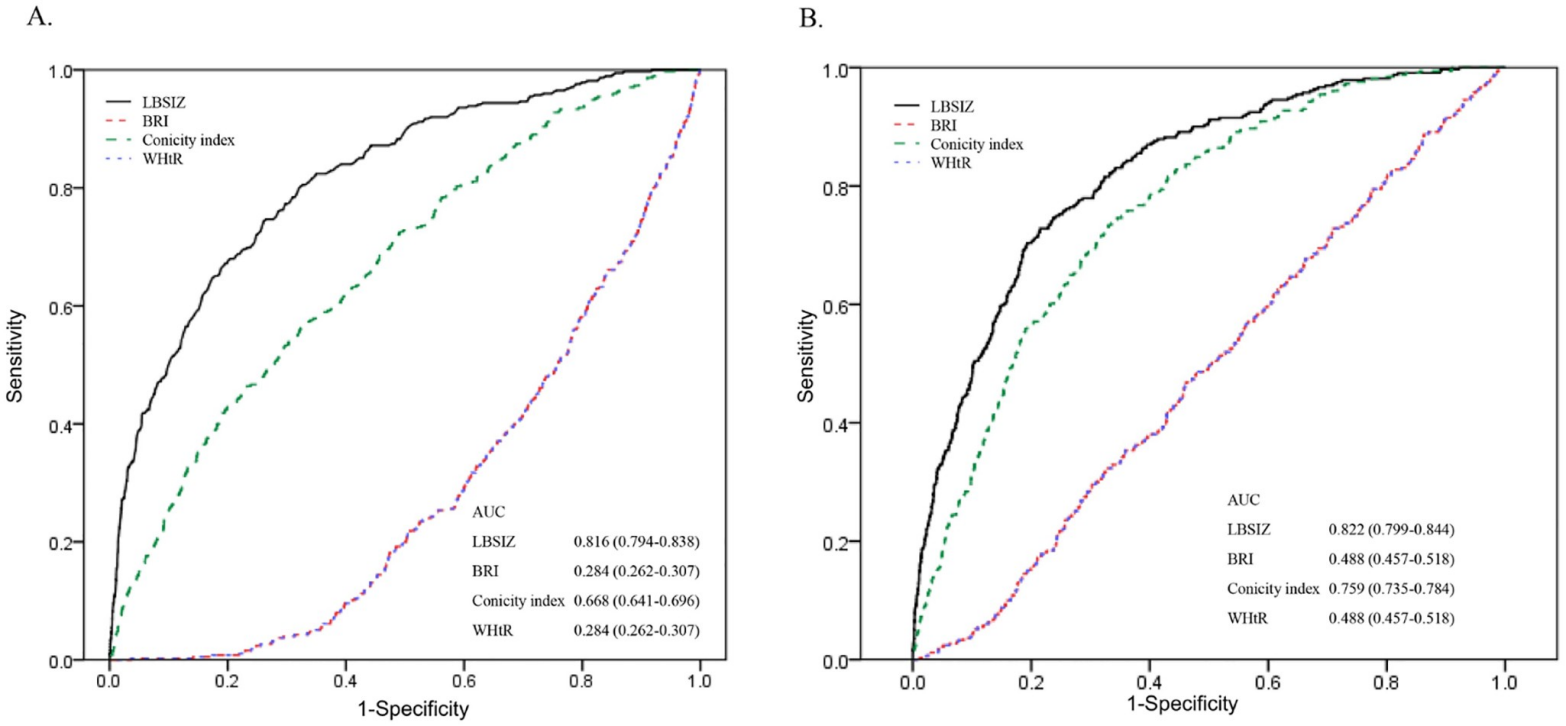
Receiver operating characteristic curves for sarcopenia according to obesity parameters. A. U.S. NHANES; B. KNHANES. LBSIZ, z-score of the log-transformed A Body Shape Index; BRI, Body Roundness Index; WHtR, waist-to-height ratio.

**Fig 3:**
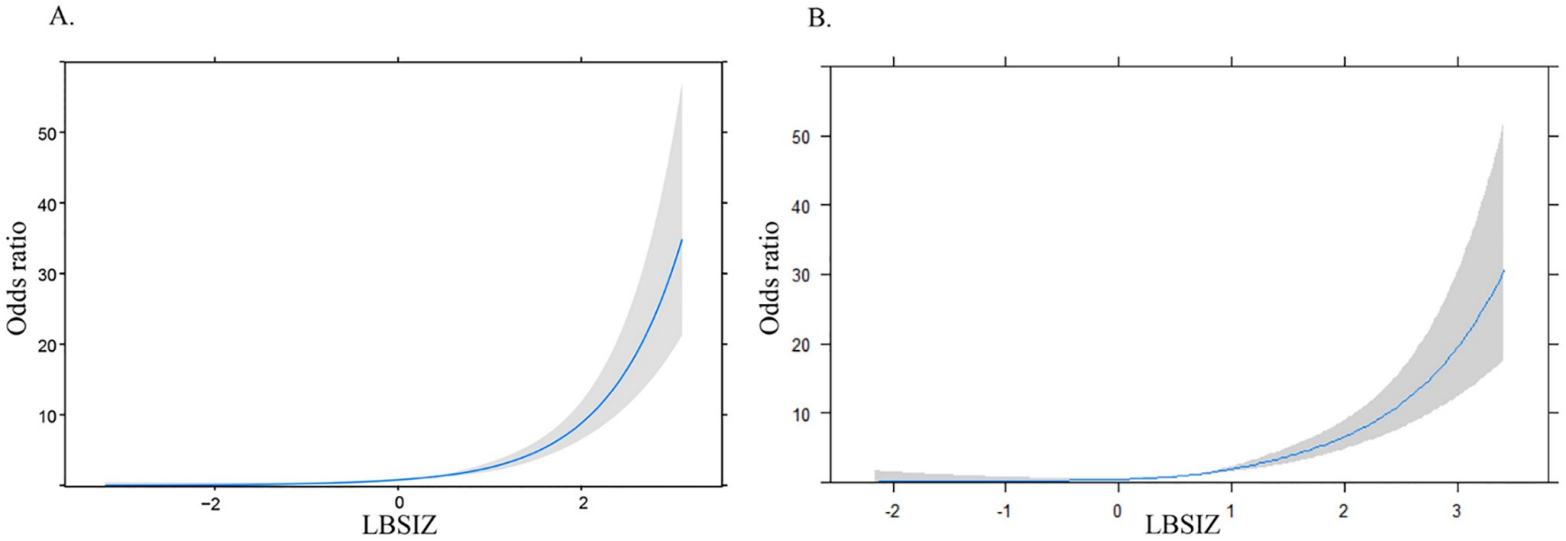
Relationship between LBSIZ and the odds ratio for sarcopenia according to sex. A. U.S. NHANES; B. KNHANES. Adjusted for age and sex. LBSIZ, z-score of the log-transformed A Body Shape Index.

Fig 2 shows the ROC curves for sarcopenia according to the obesity indices. In the U.S. NHANES, the overall AUC of the LBSIZ for sarcopenia was 0.816 (95% CI: 0.794–0.838), which was significantly higher than that of the BRI (p < 0.001), conicity index (p < 0.001), and WHtR (p<0.001). In the KNHANES, the overall AUC of the LBSIZ for sarcopenia was 0.822 (95% CI: 0.799–0.844). The AUC of the LBSIZ for sarcopenia was higher than that of the BRI (p < 0.001), conicity index (p < 0.001), and WHtR (p<0.001) for KNHANES. The AUCs and the cut-off values of the LBSIZ with respect to sex, development group, and validation group are summarized in Table 4; high AUCs are observed particularly in men. The cut-off values for American men and women were 1.05 [sensitivity, 88%; specificity, 81.5%, positive predictive value (PPV), 13.6; negative predictive value (NPV), 99.5% in the subset for developing the cut-off value; sensitivity, 94.1%; specificity, 82.9%; PPV, 13.3%; NPV, 99.8% in the subset for validation] and 0.45 (sensitivity, 77.1%; specificity, 70.6%; PPV, 15.2%; NPV, 97.8% in the subset for developing the cut-off value; sensitivity, 76.9%; specificity, 70.1%; PPV, 11.6%; NPV, 98.3% in the subset for validation), respectively. The cut-off values for Korean men and women were 1.15 (sensitivity, 77.5%; specificity, 77.1%; PPV, 24.4%; NPV, 97.3% in the subset for developing the cut-off value; sensitivity, 92.3%; specificity, 77.2%; PPV, 22.4%; NPV, 99.3% in the subset for validation) and 0.95 (sensitivity, 74.3%; specificity, 69.3%; PPV, 18.1%; NPV, 96.7% in the subset for developing the cut-off value; sensitivity, 73.6%; specificity, 66.4%; PPV, 9.6%; NPV, 98.1% in the subset for validation), respectively (Table 4). The overall ORs for sarcopenia according to the estimated LBSIZ cut-off values were 21.33 (95% CI, 10.03–45.33) for American men, 4.97 (95% CI, 3.67–6.74) for American women, 9.92 (95% CI, 6.13–16.06) for Korean men, and 4.32 (95% CI, 2.98–6.25) for Korean women, thereby highlighting that LBSIZ was more strongly predictive of sarcopenia in men compared with women (Table 4). The RCS regression plot indicated that ORs for sarcopenia rapidly increased with an increase in the LBSIZ cut-off value (Fig 3).

**Table 4:** Cut-off values of LBSIZ and their corresponding ORs for sarcopenia.

Group	Area under curve	Cut-off value	Sensitivity	Specificity	Positive predictive value	Negative Predictive Value	Age adjusted OR for sarcopenia
US NHANES							
Men							
1999–2006	0.933 (0.913–0.954)		92.1	80.2	13.5	99.7	21.33 (10.03–45.33)
2003–2006*	0.922 (0.889–0.955)	1.05	88.0	81.5	13.6	99.5	9.84 (3.86–25.08)
1999–2002^†^	0.945 (0.924–0.967)		94.1	82.9	13.3	99.8	35.58 (4.62–274.07)
Women							
1999–2006	0.806 (0.782–0.831)		77.0	70.3	13.3	98.1	4.97 (3.67–6.74)
2003–2006*	0.807 (0.776–0.839)	0.45	77.1	70.6	15.2	97.8	4.91 (3.26–7.39)
1999–2002^†^	0.805 (0.767–0.843)		76.9	70.1	11.6	98.3	4.96 (3.15–7.80)
KNHANES							
Men							
2008–2011	0.881 (0.855–0.906)		84.8	77.1	23.3	98.4	9.92 (6.13–16.06)
2010–2012*	0.849 (0.807–0.891)	1.15	77.5	77.1	24.4	97.3	6.80 (3.72–12.42)
2008–2009^†^	0.911 (0.882–0.939)		92.3	77.2	22.4	99.3	19.30 (7.92–46.72)
Women							
2008–2011	0.773 (0.739–0.808)		74.0	67.6	13.2	97.5	4.32 (2.98–6.25)
2010–2012*	0.778 (0.731–0.825)	0.95	74.3	69.3	18.1	96.7	5.16 (3.17–8.40)
2008–2009^†^	0.776 (0.727–0.825)		73.6	66.4	9.6	98.1	3.63 (2.05–6.43)

*Subset for developing the cut-off points.

^†^ Subset for validating the cut-off points.

LBSIZ, z-score of the log-transformed A Body Shape Index.

## Discussion

This nationwide population-based study was the first to show that the LBSIZ index is a powerful and reliable indicator of low muscle mass in populations with abdominal obesity. Furthermore, muscle mass was negatively associated with the LBSIZ and conicity indices but was positively associated with the BRI and WHtR. Notably, the LBSIZ index had the largest AUC in both U.S. and Korean populations with abdominal obesity. Moreover, after the analysis with respect to sex, the LBSIZ index maintained superior relationships with sarcopenia than did the conicity index, BRI, and WHtR in both populations.

In our study, LBSIZ, the modified form of the ABSI, was the most appropriate screening tool for body shape associated with sarcopenia. Previously, the usefulness of the ABSI as a cardiometabolic risk and mortality indicator was suggested for various populations [36–39]. We documented that the LBSIZ was a powerful risk factor of CVD, which could predict CVD risk better than the WC and BMI in both American and Korean populations [16, 33]. A meta-analysis that included 38 studies revealed that an increase of one SD for the ABSI was associated with an increased risk of 13%, 35%, 21%, and 55% for hypertension, type 2 diabetes, CVD, and all-cause mortality, respectively [40]. They showed that ABSI was superior to WC and BMI for the prediction of all-cause mortality [40]. Additionally, in Chinese males, the ABSI was the best indicator for the prediction of the risk of coronary heart disease that used the Framingham risk score among other anthropometric indicators, which included the BMI, WC, WHR, WHtR, BRI, abdominal volume index, and the body adiposity index [36]. Similarly, in Spanish Caucasian men, the ABSI was identified as a better anthropometric index for cardiovascular risk than BMI and WHR [37]. Moreover, in the American population, the combined assessment of BMI and ABSI provides better estimates of the risk of cardiometabolic disease than does the standard use of BMI or WC alone [38]. In particular, participants with a high ABSI had proportionally increased risks of premature mortality, whereas those with either low or high BMI had an increased risk of mortality compared with those with a median BMI [39]. Furthermore, the ABSI had a negative association with limb lean mass (*r* = -0.26) and a positive association with trunk fat mass (*r* = +0.45), calculated using DXA [39]. The enhanced risk of mortality assessed using the ABSI compared with that using BMI or WC would have been affected by low muscle mass [39]. A previous study also demonstrated that increased ABSI, similar to sarcopenic obesity, was associated with decreased fat-free mass and increased fat mass in men, whereas WC and BMI could not differentiate between fat-free mass and fat mass [41]. Especially, with an increase in the BMI, the strength of the inverse association of the ABSI with fat-free mass increased [41]. In this respect, Dhana et al. suggested ABSI as a potential marker for sarcopenic obesity [41].

Because sarcopenia is associated with major unhealthy outcomes, it requires easy, reliable, effective, and validated screening tools that are easily applicable for community-dwelling older adults. Therefore, the revised EWGSOP2 recommended screening tools to identify and assess sarcopenia in clinical practice before obtaining confirmation with DXA, BIA, MRI, or CT [28]. Generally, screening tools for sarcopenia are questionnaires, performance tests, and anthropometric measurements. Until now, questionnaire-based methods, such as Strength, Assistance with walking, Rise from a chair, Climb stairs and Falls, have been the most widely studied and recommended [42]. However, previous pooled analyses showed that the possibility of a missed diagnosis for sarcopenia was increased because of the low sensitivity and high specificity of the questionnaires [42, 43]. To overcome these limitations, it was suggested that an anthropometric measure that could serve as a diagnostic proxy for muscle mass be added to the screening tool for sarcopenia [42].

Meanwhile, limited studies have assessed the risk of sarcopenia using anthropometric parameters. The calf circumference, mid-arm muscle circumference, and skin fold thickness were previously introduced as diagnostic substitutes for sarcopenia in the absence of other available diagnostic methods for muscle quantity, and they reflect survival and physical performance in older people [22, 23, 28]. However, the determination of anthropometric measurements that assess the whole body is necessary because calf circumference and mid-arm circumference may be influenced by peripheral vascular disease and peripheral edema, which are frequently accompanied by aging. Visvanathan et al. recently developed an anthropometric prediction equation (PE) that used weight, BMI, age, and sex [44]. For Austrians aged 65 years and older, they reported the AUCs for low muscle mass that used PE compared with those that used DXA were 0.854 (CI 0.816–0.891) in men and 0.791 (CI 0.738–0.843) in women [45]. Furthermore, their PEs together with grip strength showed good discriminatory power as a valuable rule-out tool for sarcopenia in primary and aged care settings, and decreased the financial cost by reducing the number of DXA assessments [45]. Nevertheless, the outcome was regarded controversial because of the lower prevalence of sarcopenia (n = 73) in the cohort, and no representation of community-dwelling individuals because the included participants resided in hospitals or residential care facilities [45]. In addition, Goodman et al. created a simple screening model by parsimonious logistic regression based on age and BMI to identify low muscle mass with NHANES data from 1999 to 2004. They included 374 (27.5%) women and 551 (39.7%) men combined with low muscle mass, defined as 1 SD below the mean of a younger reference population that was calculated based on DXA; the AUCs were >0.88 in both groups of women and men [46]. In a study of Japanese people aged 65 years or older, Ishii et al. reported a score chart for the estimated probability of sarcopenia that included variables of age, calf circumference, and grip strength, and compared it with the definition of sarcopenia according to the EWGSOP that was measured with the BIA [47]. This model was evaluated as a valuable screening tool because of its accurate sarcopenia screening model (sensitivity vs. specificity: 84.9% vs. 88.2% in men and 75.5% vs. 92.0% in women), which used easily obtained variables that included muscle mass and physical performance [47]. However, the relationship between these anthropometric parameters and the risk of sarcopenia was evaluated in older participants, regardless of their abdominal obesity. When considering the adverse outcomes, amplified by the combination of abdominal obesity and low muscle mass, representative anthropometric tools are needed to predict sarcopenic obesity.

In this study, low muscle mass was defined as cut-offs of the ASMI according to the EWGSOP2 and AWGS. Previously, it was suggested that body size was an important factor for the establishment of cut-off points for low muscle mass because of the powerful correlation between body size and muscle mass. Walowski et al. showed that the cut-off and average values for the skeletal muscle mass index rose with an increase in BMI classification for healthy Caucasian men and women [48]. Krzyminska-Siemaszko et al. reported that a general increase in BMI was connected to an increase of the cut-off point and mean for the appendicular lean mass index in Polish participants [49]. Therefore, muscle quantity needs to be corrected with parameters that consider body size. Although there are no fixed parameters because of a lack of study consistency to date [28], the AWGS recommends using the ASMI height-adjusted skeletal muscle mass instead of weight-adjusted or BMI-adjusted skeletal muscle mass. [29].

In our study with two different populations, LBSIZ was inversely correlated with ASMI and positively correlated with WC. The optimal LBSIZ cut-off point was higher in the Korean population (men, 1.15; women, 0.95) than in the American population (men, 1.05; women, 0.45). LBSIZ had a good sensitivity (77.1–88.0%) and specificity (70.6–81.5%) for American women and men with abdominal obesity, and had a good sensitivity (74.3–77.5%) and specificity (69.3–77.1%) for Korean women and men with abdominal obesity. Additionally, consistent results were verified when we applied each LBSIZ cut-off points to the NHANES and KNHANES validation dataset. The high sensitivity and NPV of the LBSIZ implied that LBSIZ might be a good screening tool to identify sarcopenia in populations with abdominal obesity. Although we could not provide the exact reason for these race-dependent differences in cut-off values, it may be because Asians have higher adiposity than Caucasians [29]. In addition, in both populations, men had higher LBSIZ cut-off points than did women. This might be because of the different criteria for abdominal obesity and sarcopenia according to sex.

This study had some limitations. First, we only investigated these relationships in the American and Korean populations with abdominal obesity; thus, the results should be confirmed in other ethnic and body shape groups. Second, because of the lack of data for hand grip, gait speed, and for the balance, and chair stand tests, we could not evaluate other sarcopenia parameters such as muscle strength or physical performance. Third, WC and body composition were measured by different methods in the NHANES and KNHANES. Therefore, careful interpretation is necessary for the comparison of the NHANES and KNHANES. Nevertheless, our study had unique strengths. This study used credible and standardized databases that were created by the American and Korean governments, which have a huge sample size and extensive information, and include medication, laboratory, and medical diagnosis data. Moreover, we confirmed the muscle quantity or quality using DXA, which is recommended as the standard method for the definition of sarcopenia. Finally, despite the differences in the race and baseline characteristics of the American and Korean populations, the relationship between body shape indices and muscle mass were meaningfully reflected with a similar pattern to the data.

## Conclusions

We found that increased LBSIZ could function as a reliable and cost-effective screening tool for the assessment of low muscle mass, and thus potentially improve individualized risk assessments compared with previous studies of body shape indices in the American and Korean populations with abdominal obesity. Further studies are required to generalize our results to other ethnic population groups and to ensure the performance of LBSIZ together with muscle strength indicators, which include gait speed, hand grip, and the chair stand test, as a screening tool for sarcopenia.
